# How I do it: Image-based robot-assisted deep brain stimulation under general anesthesia

**DOI:** 10.1007/s00701-026-06888-2

**Published:** 2026-05-07

**Authors:** Pedro Roldan, Alvaro Bedoya, Alejandra Mosteiro, Jordi Rumià

**Affiliations:** 1 Department of Surgery, Faculty of Medicine, University of Barcelona, Barcelona, Spain; 2 Department of Neurosurgery, Hospital Clínic of Barcelona, C/Villarroel 170, 08036 Barcelona, Spain

**Keywords:** DBS, Robot, General anesthesia, Workflow, Technique

## Abstract

**Background:**

Robotic assistance has been introduced in deep brain stimulation (DBS) surgery to enhance stereotactic accuracy and workflow reproducibility.

**Methods:**

We describe a step-by-step technique for robot-assisted DBS implantation using the Neuromate® system, integrating frame-based head fixation, image-based planning, robotic guidance, and intraoperative 3D imaging for accuracy verification.

**Conclusion:**

This workflow prioritizes anatomical precision, procedural reproducibility, and intraoperative verification, providing a fully image-based alternative to conventional physiological mapping strategies.

**Supplementary Information:**

The online version contains supplementary material available at 10.1007/s00701-026-06888-2.

## Relevant surgical anatomy

Accurate DBS targeting relies on patient-specific high-resolution MRI with direct anatomical visualization of deep gray matter structures. Beyond target identification, trajectory safety depends on a precise understanding of the spatial relationship between the nucleus of interest and adjacent eloquent structures. Image-based robotic guidance requires millimetric anatomical accuracy, as even small deviations may translate into suboptimal clinical response or stimulation-related adverse effects [4, 10].

For subthalamic nucleus (STN) targeting, particular attention is given to the red nucleus posteriorly and the internal capsule laterally, as millimetric deviations may result in capsular side effects and a reduced therapeutic window. Superiorly, thalamic borders are evaluated to avoid unintended placement, while inferior extension toward the substantia nigra is considered when adjusting final depth.

In globus pallidus internus (GPi) implantation, the medial border adjacent to the internal capsule is a critical landmark. Excessive lateral deviation increases the risk of capsular stimulation, whereas inferior misplacement may approach the optic tract. The anterior–posterior position is planned to remain within the motor territory of the pallidum.

For ventral intermediate nucleus (VIM) targeting, anatomical definition relies on thalamic segmentation relative to the anterior and posterior commissures. Particular attention is paid to the proximity of the internal capsule laterally and the sensory thalamic nuclei posteriorly, as misplacement may induce paresthesias or motor side effects. When tractographic data are available, the dentato-rubro-thalamic tract may serve as an adjunctive reference for trajectory refinement.

In anterior nucleus of the thalamus (ANT) implantation, ventricular anatomy is particularly relevant. The relationship with the foramen of Monro and lateral ventricle dictates trajectory planning, especially in transventricular approaches, where mechanical stability and avoidance of excessive cerebrospinal fluid loss are essential to reduce brain shift.

Targeting of the centromedian nucleus (CM) requires careful differentiation from adjacent intralaminar thalamic nuclei and consideration of its proximity to the internal medullary lamina. Medial deviation may compromise neighboring thalamic territories, while posterior extension approaches the pulvinar.

For ventral tegmental area (VTA) targeting, trajectory planning must account for its deep mesencephalic location, posterior to the mammillary bodies and medial to the substantia nigra. Close proximity to midbrain structures mandates precise depth control and avoidance of vascular perforators.

Across all targets, systematic evaluation of vascular anatomy, sulci, ventricular boundaries, and white matter pathways is mandatory during trajectory planning. A detailed understanding of individual anatomical variability remains fundamental when performing fully image-based robotic DBS [10].

## Technique

### Target definition

Common targets in our practice include the subthalamic nucleus (STN), globus pallidus internus (GPi), ventral intermediate nucleus of the thalamus (VIM), anterior nucleus of the thalamus (ANT), and ventral tegmental area (VTA). Target is selected according to the therapeutic goal.

Target selection determines the implantation instrumentation. A 1.8-mm reducer system mounted on the robotic arm is used in most cases, whereas for transventricular trajectories, such as those targeting the anterior nucleus of the thalamus, the Alpha Omega® guiding system is preferred, as the cannula provides additional mechanical guidance and trajectory stability.

Preoperative planning is image-based and relies on high-resolution MRI for direct anatomical target definition, complemented by thin-slice CT for stereotactic registration and image fusion [9, 10]. Diffusion tensor imaging and stereotactic atlases may be used as adjuncts but are not mandatory [9, 10]. Trajectories are planned to avoid sulci, ventricular transgression, vascular structures, and eloquent areas, prioritizing reproducibility over physiological mapping [8, 10].

### Operating room setup and head fixation

**Fig. 1:**
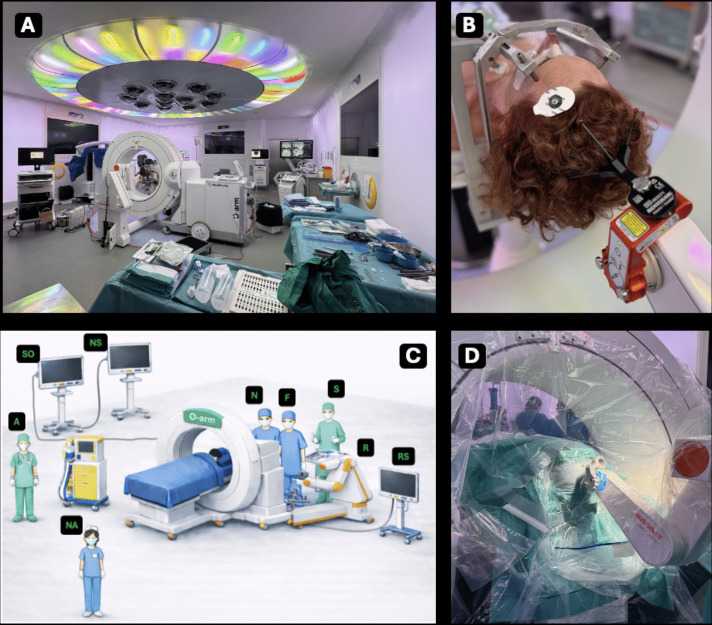
Standardized operating room setup for robot-assisted deep brain stimulation. **A** Hybrid surgical suite integrating O-arm intraoperative imaging and the Neuromate® robotic platform. **B** Leksell frame fixation coupled to the robotic arm with frontal fiducial for registration verification. **C** Structured operating room layout ensuring unobstructed robotic movement and imaging acquisition. Neurosurgeon (N), fellowship (F), nursing assistant (NA), anesthesiologist (A), scrub nurse (S), O-arm software (SO), neuronavigation system (NS), robot software (RS). **D** Sterile preparation of the robotic arm and cranial field prior to electrode implantation

The procedure is performed under general anesthesia. After completion of a standard robotic system checklist, the patient’s head is rigidly fixed to the robotic platform using a Leksell frame, not for stereotactic purposes. The patient is positioned supine with slight trunk elevation to facilitate venous drainage and reduce the risk of pneumocephalus [5]. The frame is then coupled to the Neuromate® robotic system, allowing execution of stereotactic movements with robotic precision [4, 9] (Fig. 1).

### Robotic registration and accuracy verification

**Fig. 2:**
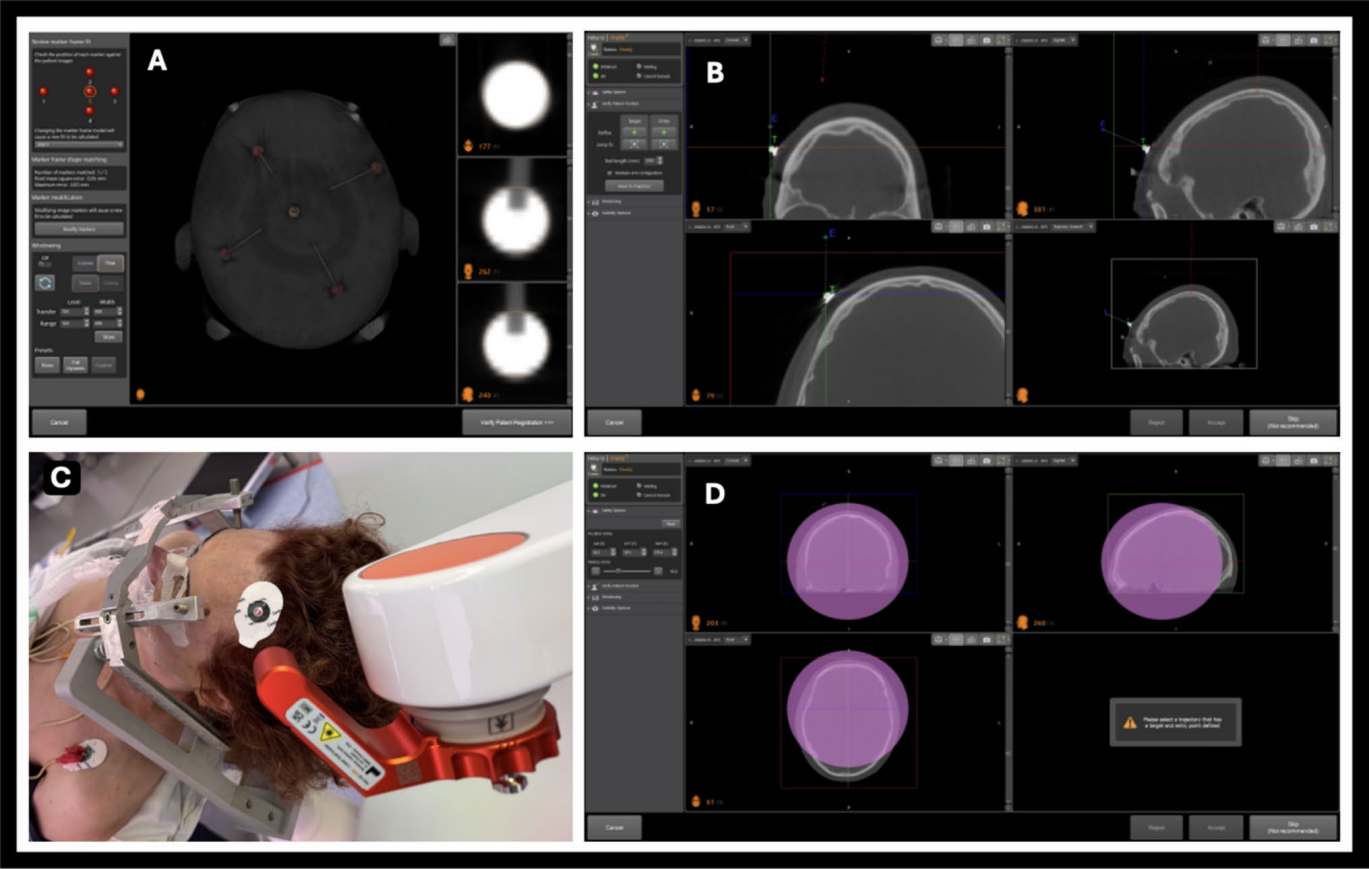
Robotic registration workflow prior to electrode implantation. **A** Virtual stereotactic reference and fiducial configuration within the planning software. **B** Multiplanar image-based verification of fiducial coordinates. **C** Frontal fiducial marker positioned for registration accuracy assessment. **D** Intraoperative 3D imaging fused with the surgical plan, a virtual safety sphere is defined to constrain robotic movements before trajectory execution

A frontal fiducial marker is placed for robotic accuracy verification. The Neuromate laser and Neurolocate system are mounted on the robotic arm, and intraoperative 3D imaging is acquired with an O-arm and fused with preoperative plan [1]. Fiducial recognition an accuracy are verified, with manual correction if required, providing a quantitative accuracy estimate [1]. A virtual safety sphere is defined to restrict robotic movements, and final system calibration is confirmed by executing a test trajectory before electrode implantation (Fig. 2).

### Electrode implantation

The O-arm is positioned in a working configuration that allows space for surgical maneuverability. Acquisition and parking positions are recorded. The patient and robotic arm are then prepared and draped in a sterile fashion.

Using the robotic guidance system, the planned skin entry point is identified and marked. A linear skin incision is performed, followed by careful subperiosteal dissection to expose the calvarium. A burr hole is drilled, and a burr-hole fixation device (Stimloc® or SureTek®) is secured in place.

The dura mater is opened sharply using a scalpel. At this stage, minimizing cerebrospinal fluid (CSF) leakage is essential to reduce pneumocephalus.

The electrode is prepared on the back table, paying special attention to the manufacturer's directional orientation before insertion [5].

The Neuromate robotic arm is then positioned according to the pre-established stereotactic coordinates. The distance to the target must be defined in the robot workstation, and this should be equal to the length of the guide canula being used. The guide cannula is advanced through the 1.8-mm reducer system along the planned trajectory. Once the cannula reaches the target depth, it is maintained in position for approximately 30–45 s before being gently withdrawn. This pause allows tissue relaxation and minimizes elastic recoil along the trajectory.

The electrode is subsequently introduced through the reducer, following the trajectory previously carved by the cannula. Correct rotational alignment of the electrode is essential when implanting directional electrodes [8].

Fibrin sealant (Tisseel®) is applied at the burr hole to promote dural sealing, reduce CSF egress, and minimize the risk of pneumocephalus. The burr-hole fixation device is then locked, ensuring stable anchoring of the electrode [5].

### Intraoperative imaging and verification of electrode position

**Fig. 3:**
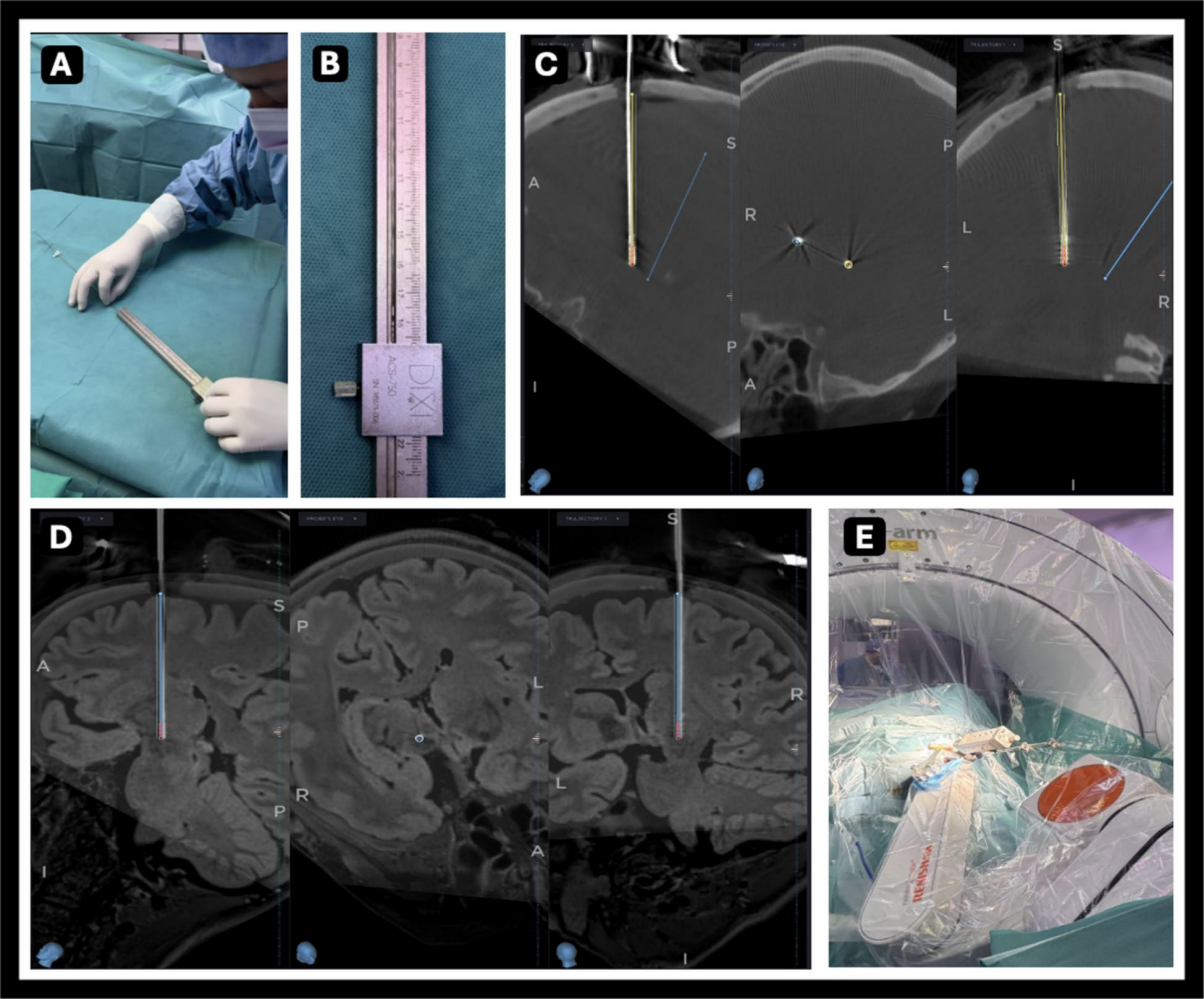
Robot-guided trajectory execution and depth confirmation. **A** Guide cannula preparation before insertion. **B** Identification of the manufacturer’s orientation marker on the directional DBS lead to ensure correct rotational alignment before implantation. **C** Intraoperative CT confirming stereotactic alignment of the trajectory. **D** MRI fusion demonstrating final anatomical target verification.** E** Sterile positioning of the robotic arm and O-arm system during trajectory execution and imaging acquisition

Intraoperative 3D imaging is acquired with the O-arm and fused with the preoperative plan to verify electrode position [2, 3, 7]. Depth and spatial alignment are assessed, and minor deviations are interpreted in relation to target anatomy. Once satisfactory positioning is confirmed, the robotic arm is disengaged, leaving the electrode secured in place (Fig. 3).

### Bilateral implantation strategy and lead tunneling

In bilateral DBS procedures, the same workflow is repeated contralaterally after confirmation of correct electrode placement on the first side, with intraoperative imaging used to detect complications. After both leads are implanted, protective caps are placed, and the electrodes are tunneled to the retroauricular region through a single incision. Burr-hole caps are secured, excess lead length is accommodated subperiosteally, and frontal wounds are closed in layers.

### Neurostimulator implantation

**Fig. 4:**
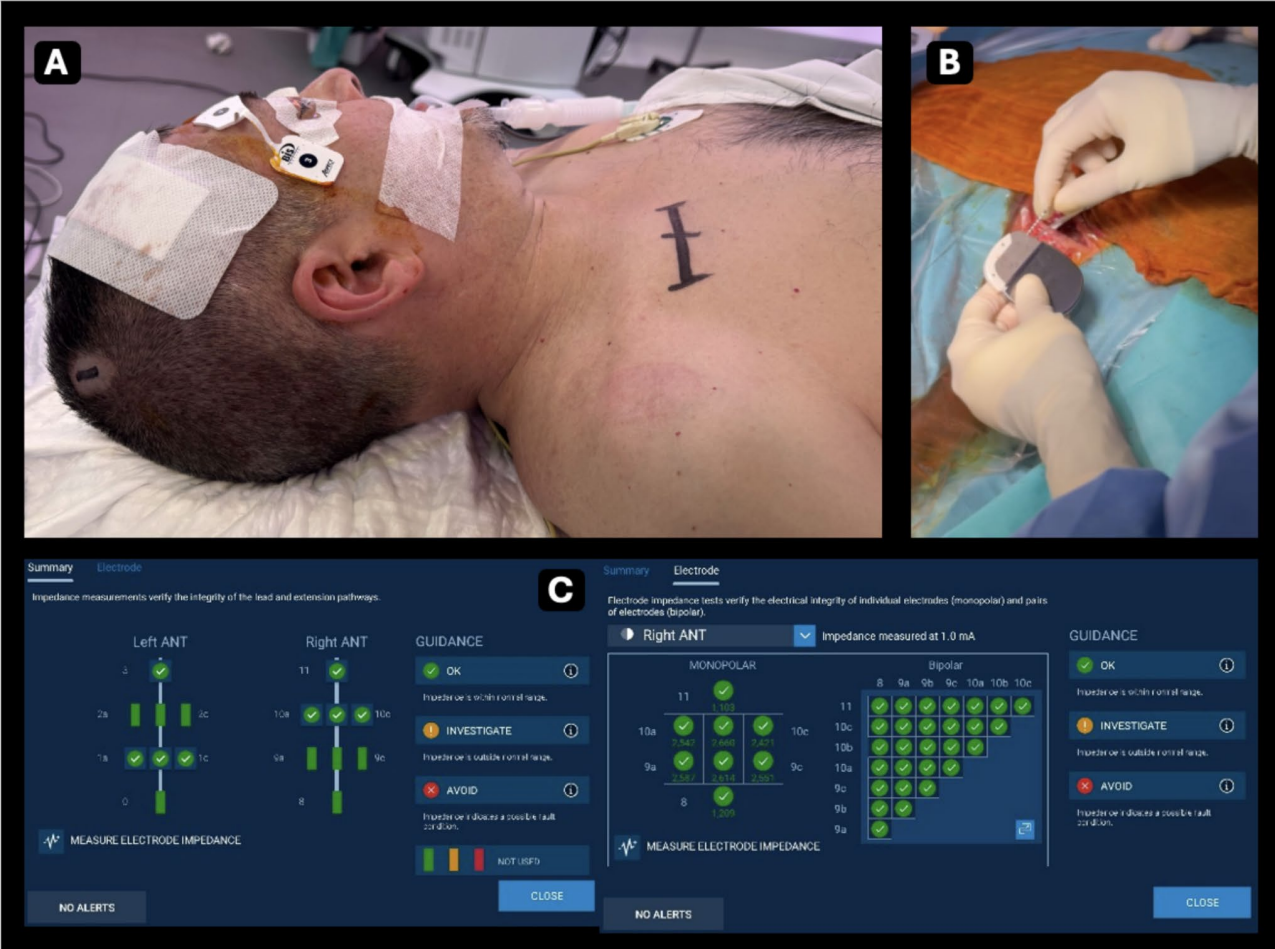
Generator implantation and electrical system validation. **A** Patient repositioned after cranial lead implantation for neurostimulator placement; parietal and infraclavicular incision sites are marked prior to neurostimulator implantation.** B** Connection of the extension cable to the neurostimulator before placement within the infraclavicular subcutaneous pocket. **C** Intraoperative impedance testing confirming electrical integrity of the leads and extension pathways prior to definitive wound closure

After cranial closure, the frame and robotic arm are disengaged, and generator implantation is performed during the same session. The patient remains supine, and the retroauricular and right infraclavicular regions are prepared. The distal electrodes are exposed retroauricular, and a subcutaneous infraclavicular pocket is created for the neurostimulator. Extension cables are tunneled and connected to the electrodes and neurostimulator. System integrity is confirmed by impedance testing, and all wounds are closed in layers (Fig. 4).

## Indications

Patient selection follows standard indications for DBS, based on multidisciplinary evaluation involving neurology, neurosurgery, neuropsychology, and psychiatry when appropriate. Given the high precision acquired with robotic-assisted electrode implantation, we have changed our institutional strategy towards a general anesthesia procedure in all DBS targets.

## Limitations

Robotic-assisted DBS involves high acquisition and maintenance costs, including the robotic platform and dedicated instrumentation, which may limit its adoption in resource-constrained settings [6].

The technique requires familiarity with robotic planning and workflow integration, and a learning curve should be expected during early adoption [4, 9].

Not all robotic systems allow seamless integration of intraoperative imaging datasets, and manual verification may still be required depending on the platform configuration [1].

## How to avoid complications

Accurate target selection determines both trajectory planning and instrumentation strategy. Transventricular or deep midline targets may require additional mechanical guidance to maintain stability. Robotic precision depends on meticulous registration and verification, as registration errors may propagate throughout the procedure and directly affect final electrode positioning.

Careful trajectory planning minimizes hemorrhagic risk by avoiding sulci, ventricular walls, and vascular structures. Prevention of excessive cerebrospinal fluid loss is essential to reduce pneumocephalus and brain shift.

When directional electrodes are used, rotational alignment must be deliberately controlled. Robotic systems ensure linear trajectory accuracy but do not inherently correct angular positioning of the lead. Verification of the manufacturer’s orientation marker prior to fixation is therefore mandatory to preserve intended segment directionality and optimize postoperative programming flexibility.

Intraoperative 3D imaging provides final confirmation of electrode position and allows early detection of complications [2, 3, 7].

## Specific perioperative considerations

Following the intervention, the patient is transferred to postoperative care unit for close monitoring for 6 h. Anti-parkinsonian medication – if indicated – is started as soon as possible. A CT scan is performed after 6 h to rule out hemorrhagic complications and as reference for final electrode position. Stimulation and programming would normally start a week after.

## Specific information to give to the patient

Major complications are intraparenchymal hematoma and device-related infection. Other potential issues to advise are bow-neck syndrome and stimulation-related adverse effects.

At discharge, patients are provided with a detailed list of precautions related to the neurostimulator (Supplementary material).

## Key points

1. Robot-assisted DBS enables reproducible image-based stereotactic trajectories.
2. Planning is individual anatomy-based using MRI–CT fusion.
3. DTI and atlases are optional adjuncts for targeting.
4. A robotic system checklist is mandatory before fixation.
5. The Leksell frame provides rigid fixation without stereotactic referencing.
6. Alpha Omega® guidance improves stability in transventricular trajectories.
7. Robotic accuracy requires fiducial-based verification with intraoperative imaging.
8. CSF loss must be minimized to prevent pneumocephalus and brain shift.
9. Directional lead orientation must be verified using the manufacturer’s marker before fixation.
10. Intraoperative 3D imaging confirms electrode position and detects complications.

## Supplementary Information

Below is the link to the electronic supplementary material.

Supplementary file1 (MP4 238024 KB)

Supplementary file2 (MP4 215990 KB)

Supplementary file3 (DOCX 15 KB)

## Data Availability

No datasets were generated or analysed during the current study.

## Abbreviations

*ANT*: Anterior nucleus of the thalamus
*CT*: Computed tomography
*CSF*: Cerebrospinal fluid
*GPi*: Globus pallidus internus
*DBS*: Deep brain stimulation
*MRI*: Magnetic resonance imaging
*STN*: Subthalamic nucleus
*VIM*: Ventral intermediate nucleus of the thalamus
*VTA*: Ventral tegmental area
*ICN*: Institut Clínic de Neurociències
